# Chronic inhibition of receptor protein tyrosine phosphatase β/ζ reduces amyloid plaque load and modulates pleiotrophin-expressing glial cells, glial-plaque interactions and genes related to amyloid beta clearance

**DOI:** 10.3389/fphar.2026.1839516

**Published:** 2026-06-19

**Authors:** Teresa Fontán-Baselga, Héctor Cañeque-Rufo, Elisa Rivera-Illades, Esther Gramage, José María Zapico, Beatriz de Pascual-Teresa, María del Pilar Ramos-Álvarez, Marta Vicente-Rodríguez, Gonzalo Herradón

**Affiliations:** 1 Department of Health and Pharmaceutical Sciences, Faculty of Pharmacy, Universidad San Pablo-CEU, CEU Universities, Urbanización Montepríncipe, Boadilla del Monte, Spain; 2 Department of Chemistry and Biochemistry, Faculty of Pharmacy, Universidad San Pablo-CEU, CEU Universities, Urbanización Montepríncipe, Boadilla del Monte, Spain

**Keywords:** Alzheimer’s disease, MY10, neurodegeneration, pleiotrophin, RPTPβ/ζ

## Abstract

Alzheimer’s disease (AD) is the most common cause of dementia. Pleiotrophin (PTN) is a neurotrophic factor relevant for central nervous system repair, neuron differentiation and survival. It is upregulated in different neuroinflammatory conditions. PTN is an endogenous inhibitor of Receptor Protein Tyrosine Phosphatase (RPTP) β/ζ. In a previous study, we showed that a short treatment with the RPTPβ/ζ inhibitor MY10 reduced amyloid beta (Aβ) plaque formation and glial activation in old APP/PS1 mice. Nevertheless, these preliminary data required new studies to prove the disease-modifying potential of RPTPβ/ζ inhibition by using younger animals and a longer treatment with MY10. Thus, we have now treated for 3 months five- to seven-month-old wild type (WT) and APP/PS1 mice with MY10. This treatment decreased Aβ plaque formation and increased the number of microglial cells in the dorsal subiculum of APP/PS1 mice. In addition, MY10 reduced the number of GFAP+, not Iba1+, cells surrounding Aβ plaques. As expected, PTN expression was upregulated in the brain of APP/PS1 compared to WT mice and it was mainly found in Iba1+ and GFAP+ cells. Interestingly, treatment with MY10 significantly decreased the expression of PTN and the number of PTN-expressing Iba1+ and GFAP+ cells. MY10 induced a significant decrease of *Mmp9* expression in the hippocampus of APP/PS1 mice, a key enzyme in AD progression. In summary, chronic inhibition of RPTPβ/ζ in APP/PS1 mice reduces Aβ plaque deposition, modulating glial-plaque interactions and the expression of specific genes including *Ptn* and its receptor.

## 1 Introduction

Dementia has become the seventh leading cause of death among all diseases, and the cases will raise up to 139 million by 2050 (Simon Long and Weidner, 2023). Alzheimer´s disease (AD) is the most common form of dementia, affecting more than 50 million people worldwide and it is estimated that cases will double every 5 years (Breijyeh and Karaman, 2020). It is becoming one of the most lethal and expensive diseases of this century (Scheltens et al., 2021).

The etiopathology and pathophysiology of AD are still unclear. AD is a slowly progressive neurodegenerative disease characterized by two histopathological hallmarks, neurofibrillary tangles of hyperphosphorylated Tau and senile plaques, composed of amyloid beta (Aβ) peptide aggregates (Zhao and Huai, 2023; Zolezzi et al., 2014). Nevertheless, there are studies that suggest that chronic neuroinflammation plays a key role in its pathophysiology as it does in other neurodegenerative diseases such as Parkinson’s disease (PD) or multiple sclerosis (Glass et al., 2010; Kwon and Koh, 2020).

In the event of damage to the central nervous system (CNS), the neuroinflammatory response is triggered. In the context of an acute damage, glial cells react undergoing conformational and functional alterations as well as secreting proinflammatory cytokines (Kwon and Koh, 2020; Calsolaro and Edison, 2016; Cavaliere and Traina, 2023). Acute neuroinflammation induces tissue repair signalling, neurogenesis and clearance of cell debris (Yu et al., 2022; Rodríguez-Zapata et al., 2024). However, in the case of an irreparable damage, neuroinflammation becomes chronic, turning into a permanent and low-grade response (Lecca et al., 2022). Glial cells remain reactive long-term, resulting in an uncontrolled neuroinflammation giving rise to neuronal loss and the progression of the disease (Glass et al., 2010; Carriba and Comella, 2015).

Pleiotrophin (PTN) is a neurotrophic factor relevant for CNS repair, neuron differentiation and survival (Herradón and Pérez-García, 2014). It is mainly expressed in the CNS during embryonic and neonatal periods, but it is also present in the adult in different organs such as the brain, testis, prostate and pancreas (Ballesteros-Pla et al., 2023). In the brain, PTN is produced by neurons, glial cells and pericytes and it is upregulated in neuroinflammatory conditions depending on the nature and intensity of the harmful stimulus. In addition, it is a relevant modulator of the neuroimmune response and neuroinflammation induced by different stimuli. Among which, the administration of drugs of abuse (Vicente-Rodríguez et al., 2016; Galán-Llario et al., 2023), ischemic damage (Fang et al., 2013), AD and PD (Levites et al., 2024; Alguacil and Herradón, 2015; Fernández-Calle et al., 2017) stand out. PTN binds to Receptor Protein Tyrosine Phosphatase β/ζ (RPTPβ/ζ, also known as PTPRZ1) inhibiting its phosphatase activity and increasing the phosphorylation of its substrates, such as Anaplastic Lymphoma Kinase (ALK) (Perez-Pinera et al., 2007) and Fyn Kinase (Pariser et al., 2005). RPTPβ/ζ is mainly expressed in the adult CNS and plays a key role in the modulation of neuroinflammation (Herradon et al., 2019; González-Castillo et al., 2014). RPTPβ/ζ is a transmembrane phosphatase with extracellular domains rich in chondroitin sulfate, which enables it to interact efficiently with heparin-binding proteins such as PTN. In addition, it contains two intracellular domains with phosphatase activity; D1 exhibits the highest catalytic activity, whereas the activity of D2 is very limited. In addition, RPTPβ/ζ is a member of the type V subfamily of RPTPs and can be localized in the cell membrane, in the cytoplasm and in the cell nucleus (Papadimitriou et al., 2016). It has three known variants: PTPRZ-A, a full-length receptor form; PTPRZ-B which is a shorter form and PTPRZ-S, a secretory variant of PTPRZ-A. The first two isoforms share structural characteristics like an extracellular carbonic anhydrase-like domain; a fibronectin type III-like domain, a transmembrane region, two tyrosine phosphatase catalytic domains and a C-terminal PDZ-binding motif (Papadimitriou et al., 2016).

In this regard, to further study the role of the PTN/RPTPβ/ζ signalling pathway, we designed and synthesized MY10, a blood-brain barrier (BBB)-permeable compound that selectively binds to the intracellular domain PD1 of RPTPβ/ζ, inhibiting its phosphatase activity (Pastor et al., 2018). Previous studies from our group revealed that the inhibition of RPTPβ/ζ by MY10 in mouse models of adolescent alcohol consumption, modulated the neuroimmune responses in the hippocampus (Galán-Llario et al., 2023). Additionally, MY10-mediated inhibition of RPTPβ/ζ modulated the proinflammatory response in a sex-dependent manner (Rodríguez-Zapata et al., 2023) and it enhanced microglial response to lipopolysaccharide (LPS) (Fernández-Calle et al., 2020). The APP/PS1 AD model is a double transgenic mouse with a chimeric mouse/human amyloid precursor protein and a mutant human presenilin 1 (PS1). Both mutations are associated with early-onset AD (Carrera et al., 2013). Recently, as a proof of concept, we did an intermittent treatment with MY10 for only 15 days in old APP/PS1 mice, observing a significant decrease in senile plaques in the dorsal subiculum and decreased neuroinflammation in the hippocampus (Fontán-Baselga et al., 2024). The hippocampal formation, which includes several key structures like the dentate gyrus, the *cornu ammonis* or the subiculum, is a brain structure key in memory, spatial navigation and learning processes (O'Mara et al., 2001). Moreover, these regions are the main affected in AD (Ye et al., 2024; Van Hoesen and Hyman, 1990).

To explore this novel therapeutic agent through a more translational design, we have now performed a chronic treatment with MY10 in APP/PS1 mice at earlier stages of the disease, obtaining data that indicates the disease-modifying potential of RPTPβ/ζ inhibition.

## 2 Materials and methods

### 2.1 Animals

C57BL/6 WT and heterozygous APPswe/PS1De9 (APP/PS1) double-transgenic female and male mice with a C57BL/6 background were used in this study. APP/PS1 mice were originally obtained from The Jackson Laboratory (Bar Harbor, ME, United States; MMRRC strain #034829-JAX; RRID: MMRRC_034829-JAX). The colony used in this study was kindly provided by Prof. Javier de Felipe (Cajal Institute, Madrid, Spain) and maintained in-house for experimental use. Mice were divided randomly with free access to food and water and housed in a specific pathogen-free room at 22 °C ± 1 °C with 12 h light/dark cycles. All the animals were handled and maintained in accordance with the European Union Laboratory Animal Care Rules (2010/63/EU directive) and protocols were approved by the Animal Research Committee of CEU San Pablo University and by Comunidad de Madrid (PROEX 140.3/22).

### 2.2 Treatment

MY10, 4-[(4-(trifluoromethylthio) phenoxy) methyl] phenyl trifluoromethyl sulfide, is a selective inhibitor of RPTPβ/ζ which was synthesized as previously described (Pastor et al., 2018), referred in that article as molecule 10a. Five- to seven- months-old male and female WT and APP/PS1 mice were administered with MY10 (60 or 90 mg/kg, in a volume of 0.1 mL) or its vehicle (VEH; 10% dehydrated ethanol, 20% polysorbate 80, 70% PEG- 300) as a control. These doses were selected based on pharmacokinetic studies in mice, which demonstrated a brain-to-plasma ratio of 3:1 1 hour after intragastric administration of 60 mg/kg MY10 (Pastor et al., 2018; Fernández-Calle et al., 2018). Additionally, we previously reported dose-dependent effects (15–120 mg/kg) on hippocampal gene expression in mice (Galán-Llario et al., 2024). The treatment was administered via oral gavage every other day for a duration of 3 months Following the administration protocol, animals were monitored to ensure no harmful effects.

### 2.3 Tissue collection

After 3 months of treatment, animals were sacrificed (n = 5–14/group) by decapitation under CO_2_ exposure. The brains were divided in two hemispheres and were coded to ensure blinded posterior analysis. The left hemispheres were freshly collected and conserved at −80 °C and were used for molecular analysis. Right hemispheres were post-fixed in paraformaldehyde (PFA) 4% for 48 h and then incubated in sucrose 30% at 4 °C for histological studies.

### 2.4 Immunofluorescence

Fixed brain hemispheres from all the experimental groups were coronally cut at 30 μm thickness using a sliding microtome (Leica SM2010 R) and collected in phosphate buffer (PB) 1X with 0.02% azide. Immunohistochemistry studies were performed on the dorsal subiculum of the hippocampus of each animal (Bregma −2.69 mm to −3.87 mm).

Triple immunofluorescence of Aβ (Aβ; Abcam, Cambridge, United Kingdom; Ab201060; 1:1,000), PTN (PTN; Santa Cruz Biotechnology, Texas, United States; sc-74443; 1:50); Iba1 for microglia (Iba1; Abcam, Cambridge, United Kingdom; Ab5076; 1:1,000); or GFAP for astrocytes (GFAP; Thermo Fisher Scientific, Massachusetts, United States; PA110004; 1:1,000), or NeuN for neurons (NeuN; Synaptic Systems, Gottingen, Germany; SYSY266006; 1:200) were performed as previously described in Fontán-Baselga et al. (2024).

Free floating sections were washed 3 times for 5 min with PBS and blocked with 5% bovine serum albumin (BSA) in PBS-X (0.2%; Triton X-100) for 40 min. Then, sections were again washed 3 times with PBS and then they were incubated overnight at 4 °C with a mix of primary antibodies on blocking solution (see Supplementary Table S1). Then they were incubated by the appropriate Alexa-conjugated secondary antibodies in PBS-X (0.2%; Triton X-100) (see Supplementary Table S1) for 2 h. Subsequently, the sections were counterstained for 5 min with 4′,6-diamidino-2-phenylindole (DAPI; 1:5,000) in PBS and then washed 3 times with PBS before being mounted with Fluoromont® mounting medium (Thermo Fisher Scientific; Massachusetts, United States; 00-4958-02).

Imaging was performed using a Leica DMI8 fluorescence confocal microscope. For relative quantification of immunofluorescence, one 380 μm × 380 µm photomicrograph containing series of ∼0.4 µm deep Z stacks, corresponding to ∼12 optical sections at 63X fields from the three fluorescence channels were captured from a dorsal subiculum area of the hippocampus (Bregma −2.69 mm to −3.87 mm) per animal (refraction index, 1.518). The images were captured using the LAS X Core software (Leica Microsystems, Wetzlar, Germany; offline version).

### 2.5 Image analysis

For each photomicrograph, the total number of Aβ count and Aβ % Area; GFAP+ cells and % Area; Iba1+ cells; NeuN+ cells; GFAP+ cells surrounding Aβ plaques; Iba1+ cells surrounding Aβ plaques and NeuN+ cells surrounding Aβ plaques; PTN % Area; GFAP+/PTN+ cells (astrocytes expressing PTN); Iba1+/PTN+ cells (microglial/macrophages expressing PTN); NeuN+/PTN+ cells (neurons expressing PTN) were counted in the subiculum of every subject using ImageJ/Fiji software (NIH, Bethesda, MD, United States, Version 1.50 f) as described in Fontán-Baselga et al. (2024).

### 2.6 Quantitative real-time PCR

The remaining hemispheres from all experimental groups were dissected with the Mouse Brain matrix (Agnthos, Sweeden, 69-2165-1) to obtain the whole hippocampus. RNA isolation, First-strand cDNA synthesis and quantitative real-time PCR (qPCR) analysis were performed as previously described (Fontán-Baselga et al., 2024; Cañeque-Rufo et al., 2023). Briefly, the hippocampal RNA was isolated using the phenol-chloroform method with TRIzol® reagent (Ambion). Then, total RNA was purified using the Total RNA Isolation Kit (Nzytech, Lisbon, Portugal) following the manufacturer protocol. RNA quantification and quality assessment were performed using a NanoVue Plus spectrophotometer (GE Healthcare, England). RNA purity was evaluated according to the 260/280 and 260/230 ratios. Total RNA was subsequently stored at −80 °C until use.

First-strand cDNA was synthesized using the first-strand cDNA Synthesis Kit (Nzytech) following the manufacturer protocol, and 1 μg of RNA were reverse-transcribed to DNA. qPCR analysis was performed using the SYBR green method (Quantimix Easy kit, Biotools, Madrid, Spain) in a CFX Opus 96 Real-Time System (Bio-Rad, Hercules, CA, United States). The relative expression of each gene was normalized using *Rpl13* and *Hprt* as housekeeping genes, and the data was analyzed by the Livak method. The results are expressed as fold-change versus VEH-treated animals. The primer sequences used, experimental conditions and additional information are shown in Supplementary Table S2.

### 2.7 Statistical analysis

Statistical analyses were done using Graph-Pad Prism program version 8 (San Diego, CA, United States). The Shapiro-Wilk test was to assess the normality of the sample distribution. Data was analysed using a three- or a two-way ANOVA with genotype, treatment and sex as variables as appropriate. When relevant, to better dissect the effect of each variable, we used either an unpaired t-test, a two- or a one-way ANOVA, excluding the non-significant variable if the three- or the two-way ANOVA results allowed it. Significant differences were analyzed by a Bonferroni’s *Post-hoc* only when the interaction between the variables were significant in the case of three- and two-way ANOVA and always in the case of one-way ANOVA. Significant differences revealed by three-, two-way ANOVAs and *post hoc* analysis were represented with (*) for differences in the treatment, with (#) for differences in the genotype and with (and) for differences in sex. Data are presented as mean ± standard error of the mean (S.E.M.).

## 3 Results

### 3.1 Chronic inhibition of RPTPβ/ζ with MY10 reduces Aβ plaques formation and increases microglia population in the hippocampus of APP/PS1 mice

**FIGURE 1:**
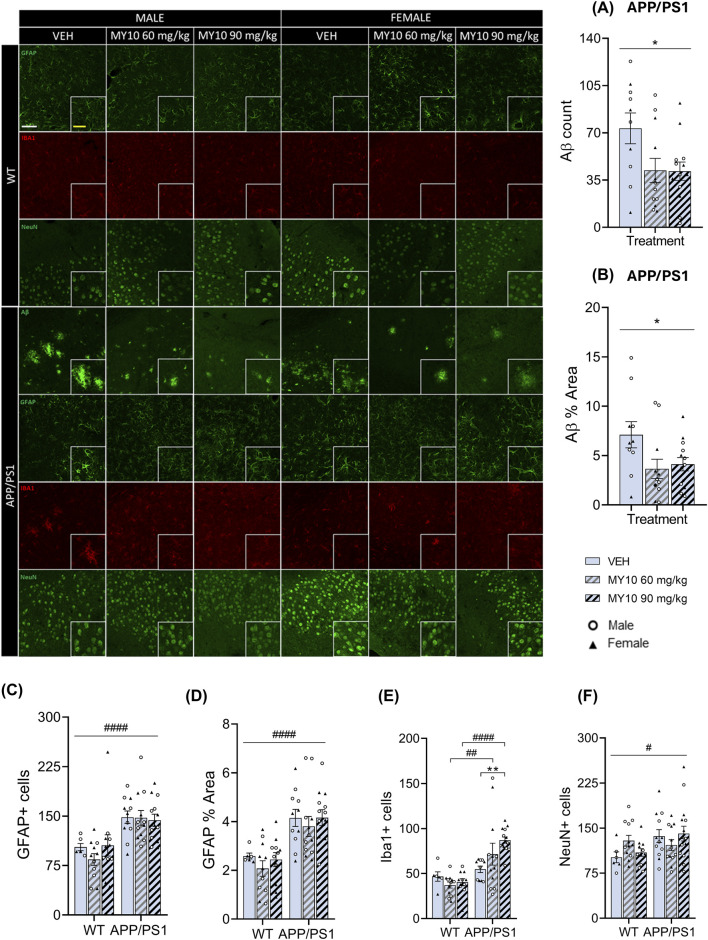
Effect of MY10 treatment on Aβ plaques, astrocytes (GFAP), microglia (Iba1) and neurons (NeuN). Representative confocal photomicrographs showing Aβ (green), GFAP (green), Iba1 (red) and NeuN (green) fluorescence from dorsal subiculum of the different experimental groups. Quantification of the number of Aβ plaques on APP/PS1 mice **(A)**, Aβ % Area on APP/PS1 mice **(B)**, number of GFAP+ cells **(C)**, GFAP % Area **(D)**, number of Iba1+ cells **(E)**, number of NeuN+ cells **(F)** per subiculum of APP/PS1 and WT male and female mice treated with Vehicle (VEH), MY10 60 mg/kg or MY10 90 mg/kg. Data are presented as mean ± SEM (n = 5–12 mice/treatment). Statistical differences between the treatments are shown by *p < 0.05; **p < 0.01 and between the genotypes by #p < 0.05; ##p < 0.01; ####p < 0.0001. White scale bar 100 µm. Yellow scale bar 50 µm.

In the present study, we aimed to investigate the effects of a prolonged treatment (3 months) with MY10 on Aβ pathology in APP/PS1 mice exclusively, as WT mice do not develop senile plaques, and glial activation during earlier stages of the disease (Figure 1). Two-way ANOVA did not reveal significant treatment or sex differences on Aβ count in APP/PS1 mice (Figure 1A; F (Breijyeh and Karaman, 2020; Fernández-Calle et al., 2020) = 3.213; p = 0.0549 (treatment); F (Simon Long and Weidner, 2023; Fernández-Calle et al., 2020) = 0.02007; p = 0.8883 (sex)). Thus, to better analyze the effect of the treatment, we performed a one-way ANOVA excluding sex variable. We found a significant effect of the treatment on Aβ count (Figure 1A; F (Breijyeh and Karaman, 2020; O'Mara et al., 2001) = 3.842; p = 0.0319) and on Aβ % area (Figure 1B; F (Breijyeh and Karaman, 2020; O'Mara et al., 2001) = 3.385; p = 0.0464), indicating that treatment with 60 and 90 mg/kg MY10 reduced Aβ plaques compared to vehicle-treated APP/PS1 mice. When we analyzed astrocytes, three-way ANOVA of numbers of GFAP+ cells only revealed significant differences between genotypes (F (1, 52) = 25.11; p < 0.0001). To better understand the effect of the variables, we performed a two-way ANOVA excluding the sex variable. We found a significant effect of the genotype (Figure 1C; F (1, 58) = 27.04; p < 0.0001), since APP/PS1 mice showed a higher population of GFAP+ cells. However, we did not observe significant effects of the treatment or the interaction between variables. On the other hand, three-way ANOVA of GFAP % area also revealed significant differences in the genotype (Figure 1D; F (1, 53) = 28.89; p < 0.0001). A two-way ANOVA was performed excluding the sex variable and we also found significant differences between genotypes (Figure 1D; F (1, 58) = 33.37; p < 0.0001). Together, the data indicates an enhanced astrogliosis in APP/PS1 mice. Moreover, three-way ANOVA revealed significant effects of the genotype in the number of Iba1+ cells (Figure 1E; F (1, 53) = 27.52; p < 0.0001) and a significant interaction between treatment and genotype (Figure 1E; F (2, 53) = 3.618; p = 0.0336). To better understand the effect of the treatment, we performed a two-way ANOVA excluding sex variable. Again, we obtained significant effects of the genotype and the interaction between variables (F (1, 59) = 29.29; p < 0.0001 (genotype); F (2, 59) = 3.862; p = 0.0265 (genotype x treatment). *Post-hoc* analysis revealed that APP/PS1 mice treated with 60, and 90 mg/kg MY10 had significantly more Iba1+ cells than their WT controls. It also showed that APP/PS1 mice treated with 90 mg/kg MY10 had significantly more Iba1+ cells compared to vehicle-treated APP/PS1 mice. Regarding NeuN+ cells, three-way ANOVA revealed a significant effect of the genotype (Figure 1F; F (1, 56) = 6.262; p = 0.0153). The two-way ANOVA performed excluding the sex variable confirmed the significant effects of the genotype (F (1, 62) = 5.023; p = 0.0286), since APP/PS1 mice showed a slightly higher population of NeuN+ cells.

### 3.2 Inhibition of RPTPβ/ζ modulates astrocytes and neuronal interactions with Aβ plaques, and attenuates the elevated PTN expression and PTN-expressing glial populations in APP/PS1 mice

**FIGURE 2:**
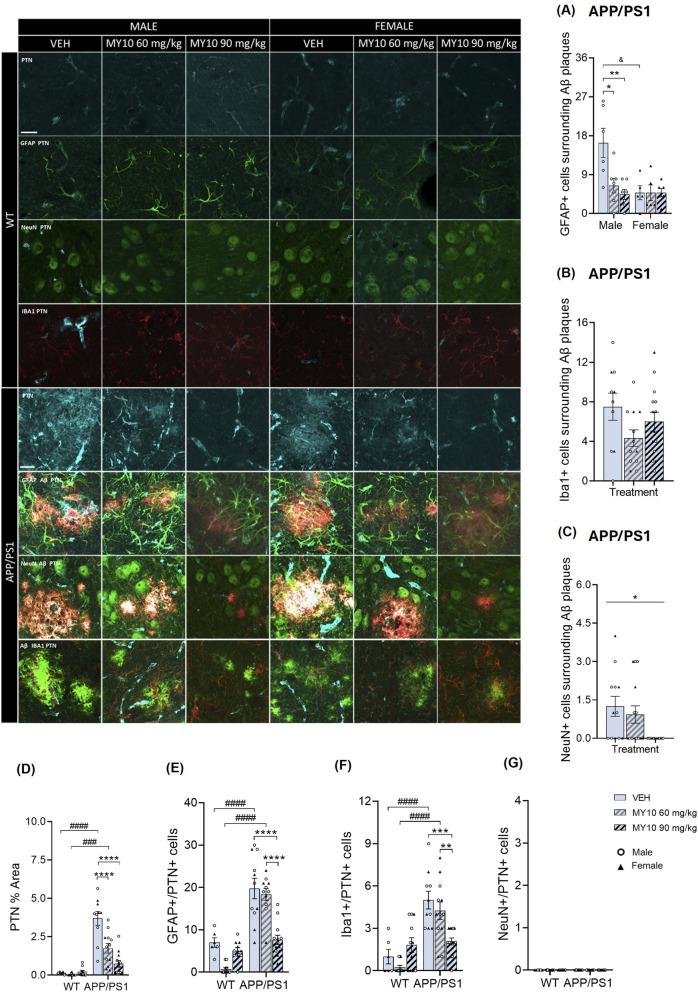
Effects of MY10 treatment on the colocalization of pleiotrophin (PTN) and Aβ plaques with astrocytes (GFAP), microglia (Iba1) and neurons (NeuN). Representative confocal photomicrographs showing Aβ, PTN, GFAP, Iba1 and NeuN fluorescence from the dorsal subiculum of the different experimental groups. Quantification of the number of GFAP+ cells surrounding Aβ plaques **(A)**, number of Iba1+ cells surrounding Aβ plaques **(B)**, number of NeuN+ cells surrounding Aβ plaques **(C)**, % area PTN+ cells **(D)**, number of GFAP+/PTN+ cells **(E)**, number of Iba1+/PTN+ cells **(F)**, number of NeuN+/PTN+ cells **(G)** per subiculum of APP/PS1 and WT male and female mice treated with Vehicle (VEH), MY10 60 mg/kg or MY10 90 mg/kg. Data are presented as mean ± SEM (n = 5–12 mice/treatment). Statistical differences between the treatments are shown by *p < 0.05; **p < 0.01; ***p < 0.001; ****p < 0.0001; between genotypes by ###p < 0.001; ####p < 0.0001; and between sex by and p < 0.05. White scale bar 100 µm.

To study the presence of PTN and the interaction of glial cells and neurons with Aβ plaques after chronic inhibition of RPTPβ/ζ, colocalization analyses were performed (Figure 2). Regarding APP/PS1 mice, two-way ANOVA showed significant effects of treatment and sex, and a significant interaction between these variables on the number of GFAP+ cells surrounding Aβ plaques (Figure 2A; F (Simon Long and Weidner, 2023; Carrera et al., 2013) = 7.246; p = 0.0115 (sex); F (Breijyeh and Karaman, 2020; Carrera et al., 2013) = 5.262; p = 0.0110 (treatment); F (Breijyeh and Karaman, 2020; Carrera et al., 2013) = 5.304; p = 0.0107 (treatment x sex)). *Post-hoc* analysis revealed that vehicle-treated male mice had more GFAP+ cells surrounding Aβ plaques than vehicle-treated female animals. Furthermore, chronic treatment with 60 and 90 mg/kg MY10 significantly reduced the number of GFAP+ cells surrounding Aβ plaques compared to vehicle-treated male APP/PS1 mice. Secondly, two-way ANOVA did not show relevant differences on the number of Iba1+ cells surrounding Aβ plaques (Figure 2B). Therefore, to better understand the effect of the treatment, we performed a one-way ANOVA excluding the sex variable which confirmed the absence of differences. We performed a two-way ANOVA of the number of NeuN+ cells surrounding Aβ plaques resulting only in significant effects of the treatment (Figure 2C; F (Breijyeh and Karaman, 2020; Fernández-Calle et al., 2018) = 3.691; p = 0.0351). One-way ANOVA excluding the sex variable showed that treatment led to a significant reduction in NeuN+ cells surrounding Aβ plaques in APP/PS1 mice treated with 90 mg/kg MY10 (F (Breijyeh and Karaman, 2020; Cañeque-Rufo et al., 2023) = 5.041; p = 0.0116). *Post-hoc* analysis revealed a significant increase of vehicle-treated mice compared to 90 mg/kg-treated ones (p = 0.014).

Interestingly, the three-way ANOVA of the PTN % area data did not detect sex differences but revealed a significant effect of both treatment with MY10, and genotype, and a significant interaction between these variables (Figure 2D; F (2, 49) = 15.40; p < 0.0001 (treatment); F (1, 49) = 83.88; p < 0.0001 (genotype); F (2, 49) = 17.31; p < 0.0001 (treatment x genotype)). Thus, we performed a two-way ANOVA excluding the sex variable observing a significant effect of the treatment, of the genotype and a significant interaction between both variables (F (2, 55) = 12.68; p < 0.0001 (treatment); F (1, 55) = 74.45; p < 0.0001 (genotype); F (2, 55) = 13.85; p < 0.0001 (treatment x genotype)). *Post-hoc* analysis revealed that both vehicle-treated and 60 mg/kg MY10-treated APP/PS1 mice exhibited higher PTN expression compared to their respective WT counterparts. On the other hand, treatment with 60 and 90 mg/kg MY10 reduced significantly the expression of PTN in APP/PS1 mice (Figure 2D). Then, we analyzed the data from colocalization assays with astrocytes (GFAP+/PTN+ cells) and with microglia (Iba1+/PTN+ cells). The three-way ANOVA revealed significant effects of the treatment, of the genotype, and a significant interaction between variables on the number of GFAP+/PTN+ cells (Figure 2E; F (2, 51) = 12.04; p < 0.0001 (treatment); F (1, 51) = 89.58; p < 0.0001 (genotype); F (2, 51) = 17.52; p < 0.0001 (treatment x genotype)). To better understand the effect of the treatment, we performed a two-way ANOVA excluding sex variable and obtained significant effects of the treatment, of the genotype and a significant interaction between both variables (F (2, 57) = 11.65; p < 0.0001 (treatment); F (1, 57) = 89.31; p < 0.0001 (genotype); F (2, 57) = 17.01; p < 0.0001 (treatment x genotype). *Post-hoc* analysis revealed that WT mice treated with VEH or MY10 (60 mg/kg) exhibited fewer GFAP+/PTN+ cells compared to APP/PS1 mice receiving the same treatments. Additionally, treatment with 90 mg/kg MY10 significantly reduced the number of GFAP+/PTN+ cells in APP/PS1 mice, compared with vehicle-treated animals. The three-way ANOVA of data from colocalization assays with microglia revealed significant effects of the genotype and a significant interaction between treatment and genotype (Figure 2F; F (1, 53) = 49.14; p < 0.0001 (genotype); F (2, 53) = 11.95; p < 0.0001 (treatment x genotype). Thus, we performed a two-way ANOVA excluding the sex variable and, again, we found a significant difference between genotypes and a significant interaction between genotype and treatment (F (1, 59) = 49.22; p < 0.0001 (genotype); F (2, 59) = 11.41; p < 0.0001 (treatment x genotype)). *Post-hoc* analysis revealed that APP/PS1 treated with VEH- or MY10 (60 mg/kg) showed a higher number of Iba1+/PTN+ cells compared to their WT counterparts. Moreover, 90 mg/kg MY10 significantly reduced the number of Iba1^+^/PTN^+^ cells compared with vehicle-treated APP/PS1 mice. Finally, we only observed a few NeuN+/PTN+ cells, which did not show any significant differences between groups (Figure 2G).

### 3.3 Chronic treatment with MY10 modulates the expression of neuroinflammation-related genes in WT mice, not in APP/PS1 mice

**FIGURE 3:**
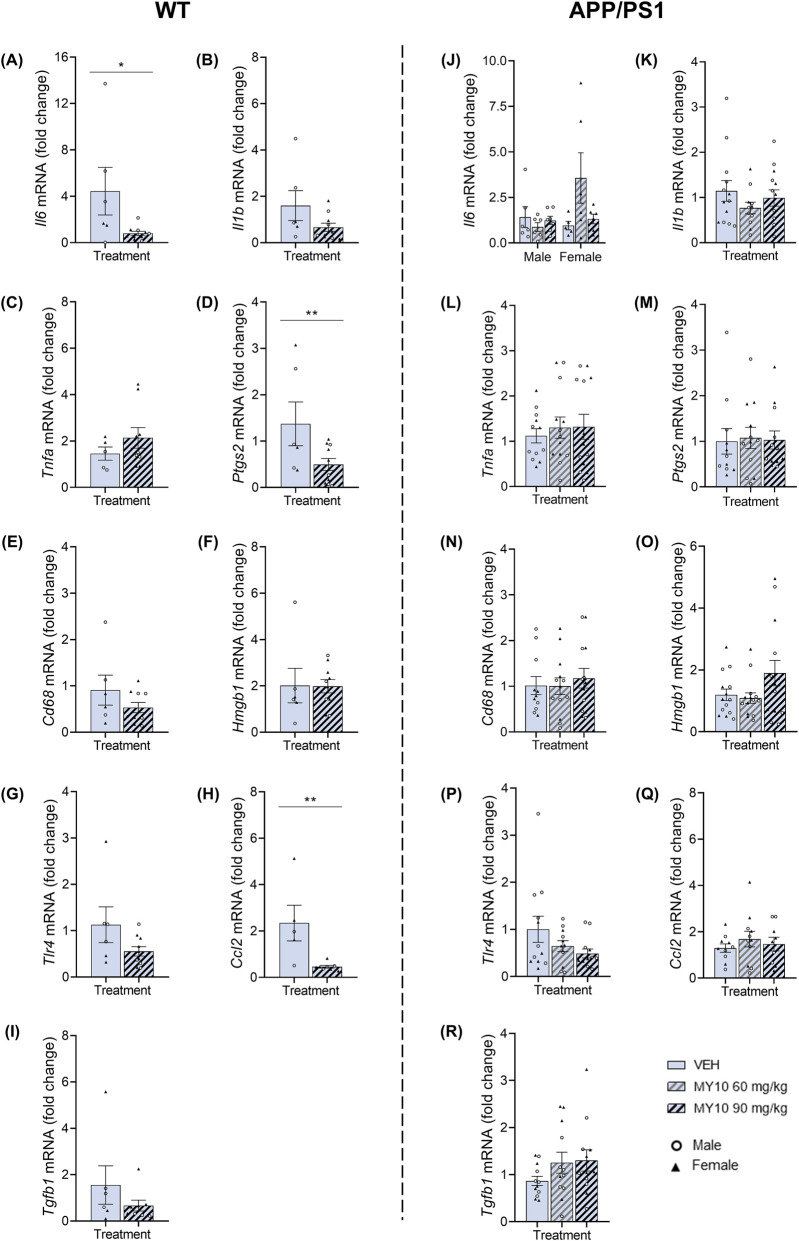
Effects of MY10 treatment on hippocampal neuroinflammatory markers. *Il6* (Interleukin 6) mRNA **(A,J)**, *Il1b* (Interleukin 1 Beta) mRNA **(B,K)**, *Tnfa* (Tumor necrosis factor Alpha) mRNA **(C,L)**, *Ptgs2* (Prostaglandin-endoperoxide synthase 2) mRNA **(D,M)**, *Cd68* (Cluster of differentiation factor 68) mRNA **(E,N)**, *Hmgb1* (High mobility group-box 1) mRNA **(F,O)**, *Tlr4* (Toll-like receptor 4) mRNA **(G,P)**, *Ccl2* (C-C motif chemokine ligand 2) mRNA **(H,Q)**, *Tgfb1* (Transforming growth factor, beta 1) mRNA **(I,R)** levels in the hippocampus of WT **(A–I)** and APP/PS1 **(J–R)** male and female mice treated with Vehicle (VEH), MY10 60 mg/kg or 90 mg/kg MY10. Data are presented as mean ± SEM (n = 5–14) mice/treatment. Statistical differences between the treatments are shown by *p < 0.05; **p < 0.01.

**FIGURE 4:**
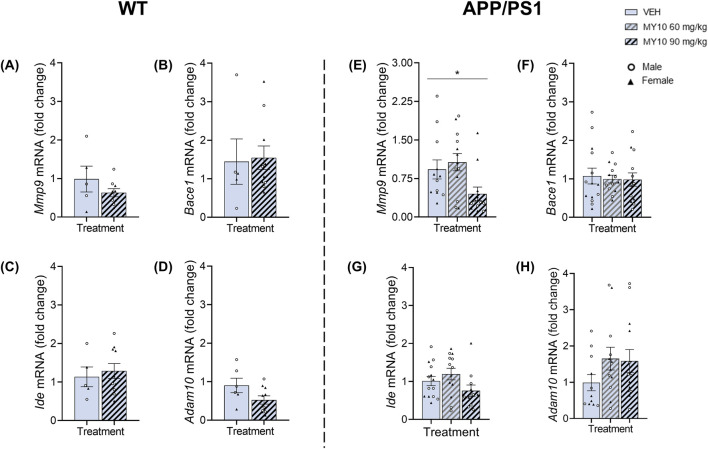
Effects of MY10 treatment on hippocampal gene expression involved in the elimination of protein aggregates. *Mmp9* (Metalloprotease 9) mRNA **(A,E)**. *Bace1* (Beta-secretase 1) mRNA **(B,F)**. *Ide* (Insulin-degrading enzyme) mRNA **(C,G)**. *Adam10* (A Disintegrin and Metalloproteinase 10) mRNA **(D,H)** in the hippocampus of WT **(A–D)** and APP/PS1 **(E–H)** male and female mice treated with Vehicle (VEH), MY10 60 mg/kg or 90 mg/kg MY10. Data are presented as mean ± SEM (n = 5–14 mice/treatment). Statistical differences between the treatments are shown by *p < 0.05.

We assessed the impact of MY10 on the expression levels of neuroinflammatory genes in the hippocampus of WT and APP/PS1 mice. Two-way ANOVA of *Il6* mRNA in WT mice only revealed significant effects of the treatment (Figure 3A; F (Simon Long and Weidner, 2023; Lecca et al., 2022) = 4.854; p = 0.0479). In contrast, treatment with MY10 did not exert significant effects on *Il6 mRNA expression* in APP/PS1 mice, but a significant interaction between treatment and sex was detected (Figure 3J; F (Breijyeh and Karaman, 2020; Carrera et al., 2013) = 3.396; p = 0.0468). Two-way ANOVA of *Ptgs2* mRNA levels in WT mice did not reveal significant main effects of the variables or an interaction between them. However, to further explore the potential impact of treatment, we performed an unpaired t-test excluding the sex factor, which showed a significant reduction of *Ptgs2* mRNA following treatment with 90 mg/kg MY10 in WT mice (Figure 3D; t (Vicente-Rodríguez et al., 2016) = 2.214; p = 0.0439). This effect was not observed in APP/PS1 mice, where *Ptgs2* expression remained unaffected by the treatment (Figure 3M). Treatment with MY10 caused a significant reduction of *Ccl2* mRNA levels in WT mice (Figure 3H; F (Simon Long and Weidner, 2023; Yu et al., 2022) = 8.731; p = 0.0144), which was not observed in APP/PS1 mice (Figure 3Q). On the other hand, statistical analyses of data from *Il1b* (Figures 3B,K); *Tnfa* (Figures 3C,L); *Cd68* (Figures 4E,N); *Hmgb1* (Figures 3F,O); *Tlr4* (Figure 3.G, P); *Tgfb1* (Figures 3I,R) mRNA levels in WT and APP/PS1 mice did not reveal significant effects of treatment or sex.

### 3.4 Chronic treatment with MY10 is associated with reduced *Mmp9* expression in APP/PS1 mice without affecting *Bace1* or *Ide*

The effect of MY10 treatment on genes involved in the elimination of protein aggregates was also studied. Firstly, we performed a two-way ANOVA of *Mmp9*, *Bace1*, *Ide* and *Adam10* mRNA levels in WT mice (Figures 4A–D), which did not reveal significant differences in any of the genes studied. Secondly, in APP/PS1 mice, two-way ANOVA of *Mmp9* mRNA expression levels showed a significant effect of treatment (Figure 4E; F (Breijyeh and Karaman, 2020; Fontán-Baselga et al., 2024) = 4.135; p = 0.0256). To further explore this finding, a one-way ANOVA excluding the sex variable was performed (Figure 4E; F (Breijyeh and Karaman, 2020; Van Hoesen and Hyman, 1990) = 3.909; p = 0.0296), confirming a significant reduction of *Mmp9* mRNA levels caused by 90 mg/kg MY10 compared to 60 mg/kg MY10 (p = 0.0332). As in the case of WT mice, we did not find significant differences in *Bace1* and *Ide* mRNA expression levels in APP/PS1 mice (Figures 4F,G). Lastly, three-way ANOVA did not show significant differences on the mRNA levels of *Adam10* (Figure 4H), which facilitates the non-amyloidogenic cleavage of APP. Two-way ANOVA excluding sex variable did not reveal significant effects either (Figure 4H), although a tendency to increase *Adam10* levels was observed in APP/PS1 mice treated with MY10. Nevertheless, it has to be noted that a 2-week treatment with MY10 (Fontán-Baselga et al., 2024) showed a significant increase in *Adam10* mRNA levels in the hippocampus of 8–10-month-old APP/PS1 mice (Supplementary Figure S1; F (Breijyeh and Karaman, 2020; Galán-Llario et al., 2024) = 4.218; p = 0.0226).

### 3.5 Chronic MY10 treatment alters the expression of *Ptn* and *Ptprz1* in APP/PS1 mice in a gene-specific manner

**FIGURE 5:**
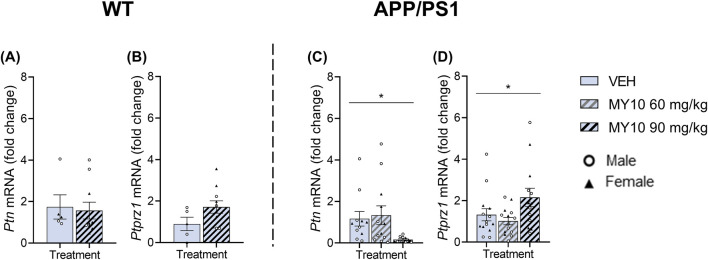
Effects of MY10 treatment on hippocampal gene expression of *Ptn* and *Ptprz1*. *Ptn* (Pleiotrophin) mRNA **(A,C)**. *Ptprz1* (Protein tyrosine phosphatase β/ζ) mRNA **(B,D)** in the hippocampus of WT **(A,B)** and APP/PS1 **(C,D)** male and female mice treated with Vehicle (VEH), MY10 60 mg/kg or 90 mg/kg MY10. Data are presented as mean ± SEM (n = 5–14 mice/treatment). Statistical differences between the treatments are shown by *p < 0.05.

We studied the effects of the treatment with MY10 on *Ptn* and its receptor *Ptprz1* (RPTPβ/ζ) mRNA levels in the hippocampus of WT and APP/PS1 mice. The two-way ANOVA of *Ptn* and *Ptprz1* mRNA levels in WT and APP/PS1 mice (Figures 5A–D) did not show significant effects of the treatment or sex. However, a one-way ANOVA excluding the sex factor revealed a significant effect of the treatment with MY10 (Figure 5C; F (Breijyeh and Karaman, 2020; Fontán-Baselga et al., 2024) = 3.433; p = 0.045), which decreased *Ptn* mRNA expression levels in APP/PS1 mice. Finally, one-way ANOVA excluding the sex factor showed a significant effect of the treatment (Figure 5D; F (Breijyeh and Karaman, 2020; Twarowski and Herbet, 2023) = 3.434; p = 0.0426), which increased *Ptprz1* mRNA expression levels in APP/PS1 mice treated with the highest dose of MY10 compared to those treated with 60 mg/kg MY10 (p = 0.0455).

## 4 Discussion

Alzheimer’s disease (AD) is the most common cause of dementia, characterized by a progressive cognitive decline and a loss of functional independence. Despite intensive research efforts, AD is still incurable (Twarowski and Herbet, 2023). In addition, its multifactorial etiology complicates the development of effective treatments, emphasizing the need for novel therapeutic targets. Our group has recently identified the PTN/RPTPβ/ζ axis as a promising novel therapeutic target for AD. In our previous study we demonstrated that short-term (14 days) treatment with the RPTPβ/ζ inhibitor MY10 significantly reduced Aβ plaque formation and glial activation in the hippocampus of 8–10-month-old male and female APP/PS1 mice (Fontán-Baselga et al., 2024). However, the limitations acknowledged in that work, i.e. only short treatment tested in old animals with amyloid pathology highly consolidated, required new studies to prove the disease-modifying potential of RPTPβ/ζ inhibition. In the present work, we examined the effects of a 3-month chronic treatment with MY10 in 5- to 7-months-old WT and APP/PS1 mice.

We observed that MY10 significantly reduced the number and size of Aβ plaques and increased microglial population in the hippocampus of APP/PS1 mice, suggesting a possible enhancement of plaque clearance mechanisms, potentially through glial activation. Interestingly, this microglial response was not accompanied by widespread changes in the expression of inflammatory genes in APP/PS1 animals, indicating that MY10 treatment may modulate the phenotype and number of microglial cells without necessarily triggering a broad pro- or anti-inflammatory shift. In this manner, treatment with MY10 induced significant decreases in some proinflammatory cytokines such as *Il6*, *Il1b*, *Ptgs2* and *Ccl2* (MCP1) in WT mice, but not in APP/PS1 mice at this stage. This may reflect that the basal immune state of WT animals may be more responsive to MY10 modulation, whereas the APP/PS1 mice inflammatory profile may be chronically stabilized and less susceptible to chronic pharmacological intervention.

The differences between the short-term study in older APP/PS1 mice and the chronic treatment in younger animals likely reflect both disease stage and treatment regimen. In the previous study, older mice with advanced pathology and established glial reactivity showed more immediate changes in glial activation following acute MY10 treatment, consistent with a highly reactive inflammatory environment (Fontán-Baselga et al., 2024). In contrast, in younger animals with less advanced pathology, chronic treatment during a more plastic disease stage may preferentially modulate glial functional states or priming rather than altering cell number or morphology. In this context, MY10 may promote a functional, potentially phagocytic microglial phenotype, facilitating amyloid clearance while preserving a relatively stable inflammatory profile. Thus, these findings are not necessarily contradictory but instead may support a context- and regimen-dependent modulation of neuroinflammatory responses by RPTPβ/ζ inhibition.

In addition, chronic MY10 treatment modulated astrocyte and neuronal interactions with Aβ plaques. Specifically, astrocyte–plaque associations were reduced by MY10 in male APP/PS1 mice, while neuronal–plaque interactions were decreased in both sexes. These changes coincided with the normalization of PTN expression and a reduction in the number of PTN-expressing glial cells induced by treatment with MY10. PTN is a neurotrophic factor involved in neural development, inflammation, and gliosis, and it has been shown to accumulate in senile plaques (Herradón and Pérez-García, 2014; Fontán-Baselga et al., 2024; Cañeque-Rufo et al., 2023; Skillbäck et al 2017). Recent findings by Levites et al. (2024) demonstrate that PTN promotes amyloid aggregation and contributes to plaque maturation. Thus, the data suggest that MY10-induced decrease of amyloid load may be related to the ability of the RPTPβ/ζ inhibitor to reduce the hippocampal expression of PTN, specially in glial cells. This is supported by the fact that treatment with MY10 caused a significant decrease of *Ptn* mRNA levels in the hippocampus of APP/PS1, in contrast to the increase in *Ptprz1* (RPTPβ/ζ) mRNA levels when treated with the higher dose of MY10.

Additionally, there are other mechanisms through which MY10 could clear Aβ plaques. Notably, MY10 reduced Matrix Metalloproteinase-9 (*Mmp9*) mRNA levels in the hippocampus. While MMP-9 has been associated with Aβ degradation, its inhibition has also been shown to attenuate plaque progression (Hernández-Guillamon et al., 2009), to reduce oxidative stress, neuronal degeneration and to enhance BBB integrity (Ringland et al., 2021). Moreover, studies have shown that higher MMP-9 plasma levels in AD patients with mild cognitive decline may promote neurodegeneration and cognitive decline (Abe et al., 2020). Interestingly, an enhanced MMP-9 activity in patients with AD and mild cognitive impairment has been shown compared to human brains with no cognitive impairment (Bruno et al., 2009). Thus, MY10-mediated downregulation of MMP-9 could contribute to its anti-amyloidogenic effects via different mechanisms that should be explored in further studies.

Furthermore, MY10 treatment tended to increase A disintegrin and metalloproteinase 10 (*Adam10*) mRNA levels in APP/PS1 mice, with a more pronounced upregulation observed in an acute treatment paradigm (Supplementary Figure S1). ADAM10 is the principal α-secretase that facilitates the non-amyloidogenic cleavage of APP, generating the neuroprotective sAPPα fragment (Elsworthy et al., 2022) and preventing Aβ formation (Zhang et al., 2011). These findings raise the hypothesis that MY10 may enhance α-secretase activity and shift APP processing towards a neuroprotective pathway. Additional studies describing the relationship between ADAM10 and the PTN/RPTPβ/ζ signalling pathway are needed.

However, there are some limitations in this study. First, although the APP/PS1 mouse model is widely used in AD research, it does not fully recapitulate the complexity of human AD. It would be interesting to study the effects of MY10 in other animal models that present the Tau pathology as well as the amyloid beta pathology. Second, while chronic MY10 treatment induced robust histological and molecular effects, additional studies including behavioral and long-term studies are required to determine the impact of MY10 on cognitive function. Third, it should be noted that mRNA expression levels do not always directly reflect protein abundance, as transcriptional changes account for only a portion of the variability observed at the protein level.

In summary, chronic inhibition of RPTPβ/ζ in APP/PS1 mice leads to reduced Aβ plaque deposition, modulation of glial–plaque interactions and altered specific gene expression patterns related to neuroinflammation and trophic signaling, particularly involving PTN and MMP9. Overall, our findings identify the PTN/RPTPβ/ζ axis as a relevant modulator of amyloid pathology and glial function, highlighting its potential as a therapeutic target that acts through multiple mechanisms, including regulation of glial phenotype and APP processing. These results also emphasize that effective disease modification in AD may require early and multi-target interventions aimed at both neuroinflammatory and amyloidogenic pathways.

## Data Availability

The raw data supporting the conclusions of this article will be made available by the authors, without undue reservation.
