# Supplementary material for: Chronic inhibition of receptor protein tyrosine phosphatase β/ζ reduces amyloid plaque load and modulates pleiotrophin-expressing glial cells, glial-plaque interactions and genes related to amyloid beta clearance
Source: Front Pharmacol. 2026 Jun 19;17:1839516. doi: 10.3389/fphar.2026.1839516 (PMC13329020; doi:10.3389/fphar.2026.1839516)
Supplement: Supplementary file 1 [file Supplementaryfile1.docx]

Supplementary Material

# Supplementary Data

## Supplementary Figures


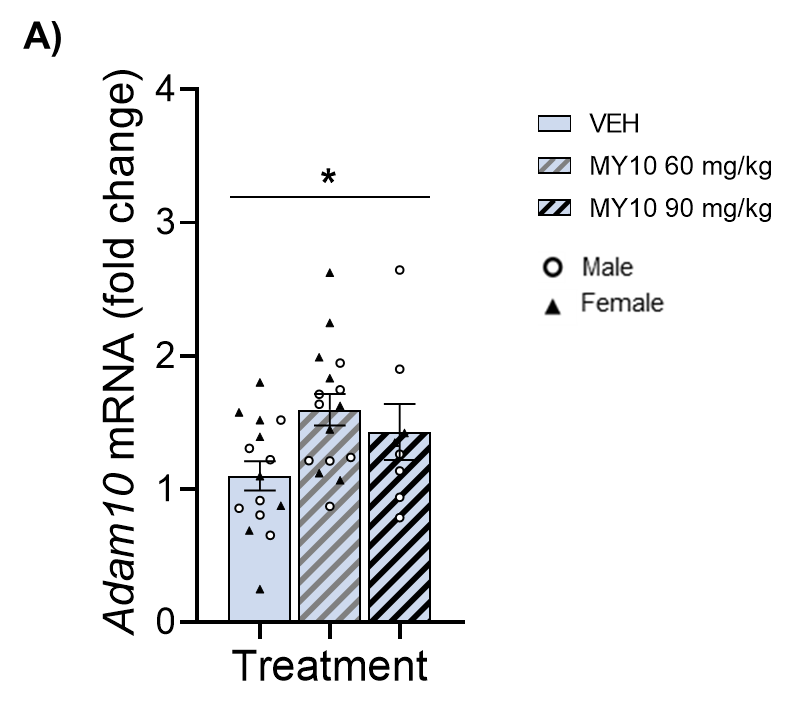


**Supplementary figure 1. Supplementary figure 1. Effects of 2-week MY10 treatment on Adam10 hippocampal mRNA levels in 8-10-months-old APP/PS1 mice.** Adam10 (A Disintegrin and Metalloproteinase 10) mRNA levels in the hippocampus of APP/PS1 male and female mice treated with Vehicle (VEH), 60 mg/kg MY10 or 90 mg/kg MY10. Data are presented as mean ± SEM (n=3-9 APP/PS1 mice/treatment). Statistical differences between the treatments are shown by *p<0.05 vs. VEH.

## Supplementary Tables

**Supplementary table 1. Primary and secondary antibodies used for immunofluorescence.** NeuN (Neuronal nuclear antigen), PTN (pleiotrophin), Aβ (amyloid beta), GFAP (glial fibrillary acidic protein), Iba1 (Ionized calcium binding adaptor molecule 1).

| **Supplementary Table 1. Primary and secondary antibodies used for immunofluorescence** | | | |
| --- | --- | --- | --- |
| **Primary antibodies** | **Dilution** | **Supplier** | **Catalog number** |
| Chicken anti-NeuN | 1:200 | Synaptic Systems | #SYSY266006 |
| Mouse anti-PTN | 1:50 | Santa Cruz | #74443 |
| Rabbit anti- Aβ | 1:1000 | Abcam | #Ab201060 |
| Chicken anti-GFAP | 1:1000 | Thermo Fisher | #PA110004 |
| Goat anti-Iba1 | 1:1000 | Abcam | #Ab5076 |
| **Secondary antibodies** | **Dilution** | **Supplier** | **Catalog number** |
| Alexa Fluor 488 anti-chicken IgY | 1:600 | Jackson Immuno | #703545155 |
| Alexa Fluor 555 anti-mouse IgG | 1:1000 | Thermo Fisher | #A21422 |
| Alexa Fluor 647 anti-rabbit IgG | 1:600 | Jackson Immuno | #711605152 |
| Alexa Fluor 647 anti-goat IgG | 1:600 | Jackson Immuno | #705605147 |
| Alexa Fluor 555 anti-mouse IgG | 1:1000 | Abcam | #Ab150106 |
| Alexa Fluor 488 anti-rabbit IgG | 1:800 | Thermo Fisher | # A21206 |

**Supplementary table 2. Primer sets used for qPCR analysis.** Cd68: Cluster of differentiation factor 68; Ide: Insulin-degrading enzyme; Il1b: Interleukin 1 beta; Il6: Interleukin 6; Mmp9: Metalloprotease 9; Ptgs2: Prostaglandin-endoperoxide synthase 2; Rpl13: Ribosomal protein L13; Bace1 (Beta-secretase 1); Hprt1: Hypoxanthine phosphoribosyltransferase 1; Tnfa: Tumor necrosis factor alpha; Hmgb1: High mobility group box 1; Ptn: Pleiotrophin; Ptprz1: Protein Tyrosine Phosphatase Receptor Type Z1; Adam10: A Disintegrin and Metalloproteinase 10; Ccl2: C-C Motif Chemokine Ligand 2; Tfgb1: Transforming Growth Factor Beta 1.
